# Development of Simple and Rapid Bead-Based Cytometric Immunoassays Using Superparamagnetic Hybrid Core–Shell Microparticles

**DOI:** 10.1021/acsmeasuresciau.4c00038

**Published:** 2024-09-17

**Authors:** Charlie Tobias, Daniel López-Puertollano, Antonio Abad-Somovilla, Josep V. Mercader, Antonio Abad-Fuentes, Knut Rurack

**Affiliations:** † Chemical and Optical Sensing Division, Bundesanstalt für Materialforschung und -prüfung (BAM), Richard-Willstätter-Str. 11, Berlin D-12489, Germany; ‡ Department of Organic Chemistry, University of Valencia, Doctor Moliner 50, Burjassot, Valencia 46100, Spain; § Institute of Agricultural Chemistry and Food Technology (IATA), Spanish Council for Scientific Research (CSIC), Av. Agustí Escardino 7, Paterna, Valencia 46980, Spain

**Keywords:** antibody-based, bead-based assay, core−shell particles, cytometry, mycotoxins

## Abstract

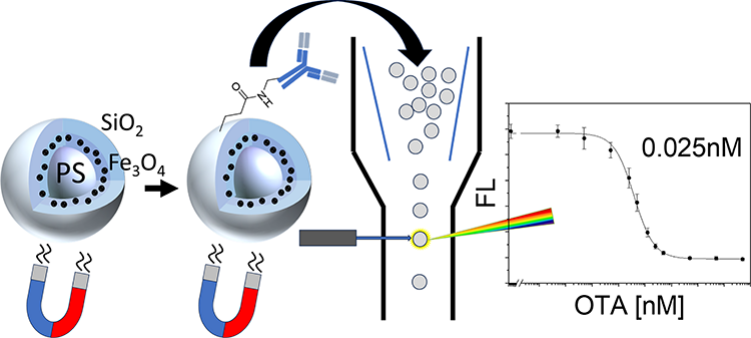

Flow cytometry-based immunoassays are valuable in biomedical research and clinical applications due to their high throughput and multianalyte capability, but their adoption in areas such as food safety and environmental monitoring is limited by long assay times and complex workflows. Rapid, simplified bead-based cytometric immunoassays are needed to make these methods viable for point-of-need applications, especially with the increasing accessibility of miniaturized cytometers. This work introduces superparamagnetic hybrid polystyrene-silica core–shell microparticles as promising alternatives to conventional polymer beads in competitive cytometric immunoassays. These beads, featuring high specificity, sensitivity, and excellent handling capabilities via magnetic separation, were evaluated with three different antibodies and binding methods, showing variations in signal intensity based on the antibody and its attachment method. The optimal performance was achieved through a secondary antibody binding approach, providing strong and consistent signals with minimal uncertainty. The optimized protocol made it possible to achieve a detection limit of 0.025 nM in a total assay time of only 15 min and was successfully used to detect ochratoxin A (OTA) in raw flour samples. This work highlights the potential of these beads as versatile tools for flow cytometry-based immunoassays, with significant implications for food safety, animal health, environmental monitoring, and clinical diagnostics.

## Introduction

Flow cytometry-based competitive immunoassays are widely used in biomedical research and clinical applications to quantify and detect different analytes in complex biological samples, since they combine the high-throughput potential of immunoassays with the multianalyte capability of liquid chromatography (LC) techniques.^1−3^ Although such approaches also hold promise in fields like food safety, animal health, and environmental monitoring, they are only slowly finding their way into these areas.^4−12^ On the one hand, this is due to the heterogeneity of the low molecular weight target analytes and the limited availability of antibodies for them, but on the other hand also to the usually rather long assay times and multistep workflows, which often take 1 h or longer due to (several) incubation steps and thus cannot even compete with LC methods in terms of time to result. However, simplicity and speed would generally be desirable if immunoanalytical methods were to make the step to point-of-need applications, for which they are generally well suited due to the advantage that robust flow cytometers become increasingly available; for instance, for resource-limited settings,^13−15^ their measurement periphery can be miniaturized^7,16^ and faster mix-and-read immunoassay formats are being developed.^9^

Central as a model analyte to the present work is ochratoxin A (OTA), a mycotoxin commonly found in grain products, coffee, cacao, grapes, and pork, posing severe health risks.^17−19^ To address these risks, the European Union (EU) has instituted maximum levels (ML) for OTA across various foodstuffs.^20^ While several methods exist for OTA analysis, immunochemical and chromatographic techniques dominate. Among the first group, enzyme-linked immunosorbent assays (ELISA) and lateral flow immunoassays (LFIA) are widely used,^21,22^ with several kits being already available on the market. Regarding chromatographic techniques, high-performance liquid chromatography (HPLC) combined with fluorescence detection, after immunoaffinity column cleanup, is the recommended approach by the European Committee for Standardization.^23^ Nevertheless, often mass spectrometry detection (MS/MS) is preferred for OTA determination at trace levels.^24,25^ While ELISA and LFIA are known for their robustness, they have drawbacks with regard to speed and handling steps (ELISA) as well as the capability for multiplexing or to process a high number of samples (LFIA), making them suitable for routine analysis of a limited set of mycotoxins and/or samples. Chromatography-based techniques are preferred for regulatory tasks that require higher accuracy, sensitivity, or a multianalyte approach, yet such analyses are slow and costly. As a potential alternative, as mentioned above, bead-based immunoassays utilizing flow cytometry offer a lot of advantages. However, only few examples have been described for OTA, using relatively heavy particles in all cases and requiring long assay times.^26−29^

As we are interested in developing cytometric and microfluidic immunoassays for on-site use, we started some time ago to develop a modular particle platform that should eventually lead to a suitable approach. The platform uses polystyrene-silica core–shell beads^30,31^ which have also been successfully used by others^32^ and are currently being commercialized.^33^ In addition, we have recently extended this platform by incorporating magnetic features.^34^ These hybrid beads consist of a polystyrene core and a silica shell in which magnetic nanoparticles are embedded, facilitating handling during washing and retention in analytical assays. The outer silica surface can be easily modified by silane chemistry so that antibodies or other molecules of interest can be attached. In addition, the particle core can be doped with fluorescent dyes, enabling use in multiplexed assays for simultaneous detection of multiple targets.^35^ Compared to particles doped with metallic nanoparticles to give them magnetic properties, these hybrid beads are lighter, resulting in slower sedimentation and making them more suitable for flow cytometry.^36−38^ These properties enable efficient analysis of large sample volumes, as heavy particles can lead to sedimentation, clogging, signal saturation, and optical interferences, which affects data quality and instrument performance in flow cytometry. When lighter particles are available as an option, they are generally preferred to avoid these problems and ensure more accurate flow cytometry measurements.^39^

In the present study, we used a competitive immunoassay approach with a fluorescent competitor to detect OTA in order to evaluate the applicability of these new materials in cytometry assays. By quantifying the amount of competitor bound to the beads, we were able to determine the OTA concentration, which allowed for precise and accurate detection without a washing or isolation step. Our second objective was to evaluate the functionalization protocol for the superparamagnetic hybrid core–shell beads. For this purpose, we investigated three different anti-OTA antibodies that had also been previously produced by our group.^40^ In addition, three methods for immobilizing the primary antibodies on the bead surface were tested, namely, direct binding of antibodies to the bead surface, binding via protein G, and binding via a secondary goat-antimouse (GAM) antibody. Finally, by employing the optimal assay configuration, we analyzed real flour samples obtained from a mill for OTA, realizing a low limit of detection in a favorable overall assay time.

## Materials and Methods

Poly(vinylpyrrolidone) (PVP10, 10 kDa, Sigma), styrene (Sigma), basic alumina (Al_2_O_3_, Brockmann I, Acros), and azo-biscyanovaleric acid (ACVA, MP Biomedicals) were used for the PVP-coated polystyrene core synthesis. FeCl_3_·6H_2_O (AppliChem) and FeCl_2_·4H_2_O (Baker) were used for the preparation of magnetic nanoparticles. Tetraethoxyorthosilicate (TEOS, Merck) and ammonia solution (NH_3_, 32%, Supelco) were used for the silica coating. (3-Aminopropyl)triethoxysilane (APTES, Aldrich) was used for amino functionalization and succinic anhydride (Merck) for later carboxylic acid functionalization. 1-Ethyl-3-[3-(dimethylamino)propyl]carbodiimide hydrochloride (EDC, Merck) and *N*-hydroxysulfosuccinimide sodium salt (sNHS, Sigma) were used for particle activation. AffiniPure Goat Anti-Mouse (GAM) IgG, Fcγ fragment specific, unconjugated secondary antibody from Jackson ImmunoResearch, and Pierce Recombinant Protein G were used for further particle functionalization. Phosphate-buffered saline (PBS, pH 7.4, 10 mM, 130 mM NaCl), 2-(*N*-morpholino)ethanesulfonic acid buffer (MES, pH 6.0, 10 mM), and bicarbonate buffer (pH 9.6, 50 mM) were prepared in Milli-Q grade water. Tris buffer was prepared using 10 mM Tris–HCl (pH 7.5), 120 mM NaCl, 20 mM CaCl_2_, and 40 mM MgCl_2_ in Milli-Q grade water. The OTA fluorescein competitor conjugate (OTA-F) and the OTA standard were purchased from Aokin. Monoclonal anti-OTA antibodies were previously produced and characterized by our group.^40^

Measurements were conducted via flow cytometry using a BD Accuri C6 instrument equipped with 488 and 640 nm lasers for excitation and included the recording of the forward scatter (FSC) and sideward scatter (SSC) signals of the particles at angles of 180 and 90°, respectively. Additionally, the fluorescence signal in the FL1 channel (488 nm, 533/30.H filter) was captured. To determine the IC_50_ values, the data obtained from the competitive assay were analyzed and plotted in the Origin software (OriginLab) using a four-parameter sigmoidal fitting. For SEM imaging, the particles were dispersed in ethanol and subjected to ultrasonication for 5 min. To prepare the samples for analysis, they were drop-casted onto conventional carbon TEM grids. Imaging of individual particles was performed using a Zeiss Supra 40 scanning electron microscope (Zeiss), equipped with a high-resolution cathode (Schottky field emitter), an Everhart–Thornley secondary electron (SE) detector, and an SE InLens detector. For transmission electron microscopy mode (TSEM or STEM-in-SEM), a dedicated “transmission” sample holder was utilized.

### Synthesis of Hybrid Particles

The superparamagnetic microparticles were prepared by a route adapted from ref (34), elaborating our earlier architecture described in refs (30,31,35) to the present one with polystyrene (PS) as the core, iron oxide (Fe_3_O_4_) nanoparticles as a magnetizable interlayer, and an outer silica shell for protection and further functionalization. The synthesis of the PS particles was carried out via dispersion polymerization by reacting a solution of 170 mg of PVP10 in 10 mL of EtOH with 1 mL of styrene, filtered through basic aluminum oxide, in a glass vial after flushing the mixture with argon for 30 min and subsequently initiating it by the addition of 0.5 mL of a solution of 105 mg of ACVA in 10 mL of MeOH, flushed with argon, under stirring at 70 °C in an argon atmosphere overnight. The resulting particles were centrifuged, washed with water and EtOH multiple times, and then dried at room temperature.

### Synthesis of Superparamagnetic Iron Oxide Nanoparticles (SPIONs)

To synthesize SPIONs, 0.465 g of FeCl_3_·6H_2_O and 0.172 g of FeCl_2_·4H_2_O were dissolved in 100 mL of Milli-Q water in a round-bottom flask. The solution was purged with argon for 20 min before slowly adding a solution of 4 g of PVP10 in 58 mL of NH_3_ solution (16%). The reaction mixture was stirred at 150 rpm for 1.5 h using a mechanical stirrer. The particles were then washed several times with water using magnetic separation and stored in a refrigerator at a concentration of approximately 3% (w/v) in Milli-Q water.

### Coating of PS Cores with SPIONs (SPION@PS)

The PS cores were coated with a layer of SPIONs by suspending 60 mg of PS cores and 2 mL of Fe_3_O_4_ particles (3% in water) in 30 mL of Milli-Q water in Falcon tubes. The coating process was carried out by placing the tubes on a rotator plate at 40 rpm for 1.5 h. Afterward, the particles were washed twice with water and once with ethanol, using magnetic separation, before drying.

### Coating of SPION@PS Particles with Silica Shell (SiO_2_@SPION@PS)

555 μL of NH_3_ solution (32%) was added to a dispersion of 60 mg of SPION@PS particles in 30 mL of EtOH and 1 mL of Milli-Q water while stirring at 150 rpm using a mechanical stirrer. Then, 555 μL of TEOS was added dropwise. The mixture was stirred overnight at 38 °C, followed by multiple washes with water and EtOH, using magnetic separation. Finally, the particles were dried at room temperature.

### Functionalization of SiO_2_@SPION@PS with Amino Groups

According to a previously reported protocol,^35^ the SiO_2_@SPION@PS particles were functionalized with amino groups by first activating the particles’ surface after suspending 20 mg of the particles in 800 μL of EtOH, adding 400 μL of 1 M HCl in EtOH, and sonicating the mixture in a sonication bath for 5 min. Afterward, the particles were washed twice with 400 μL of EtOH and redispersed in 400 μL of EtOH. For amino modification, 8 μL of APTES was added to the particle dispersion and the mixture was allowed to react in a thermomixer (800 rpm) at 40 °C overnight. Subsequently, the particles were washed three times with a mixture of EtOH:H_2_O in a 1:1 ratio, before drying in vacuum at room temperature.

### Refunctionalization of SiO_2_@SPION@PS with Carboxylic Acid Groups

To modify the surface of the materials with carboxylic acid groups for facile biomolecule attachment, 5 mg of the corresponding amino-modified particles was dispersed in 1.5 mL of absolute EtOH in a 2 mL Eppendorf tube. A solution of 30 μL 10% w/v of succinic anhydride in dimethylformamide was added to the particle dispersion, and the mixture was then allowed to react in a thermomixer (800 rpm) at 40 °C overnight. Afterward, the particles were washed three times with a mixture of EtOH:H_2_O in a 1:1 ratio. In the final step, 500 μL of ethanol was added to the particles to obtain a stock solution with a final concentration of 1% (w/v).

### Coupling of Protein G and GAM to Refunctionalized SiO_2_@SPION@PS

From the 1% (w/v) stock solutions, 10 μL of SiO_2_@SPION@PS particles was dispersed in 100 μL of MES buffer. To this particle dispersion, 80 μL of a freshly prepared solution of EDC and 160 μL of s-NHS solution, both in MES buffer and with a concentration of 1% (w/v), were added. The mixture was incubated at room temperature for 15 min, and then 50 μL of either protein G (2.4 mg mL^–1^) or GAM (2.4 mg mL^–1^) was added and incubated overnight on a rotator plate at 40 rpm and room temperature. Afterward, the particles were washed twice with 500 μL of PBS and redispersed in 200 μL of fresh PBS to reach a stock solution concentration of 0.05% (w/v).

### Direct Binding of the Primary Antibody

For the direct binding of the anti-OTA mAbs, the previously described protocol for protein G or GAM was followed. In each case, 20 μL at 1 mg mL^–1^ in PBS of each of the mAbs was used. The washing was performed as previously described for the capture protein-modified particles.

### Coupling of the Primary Antibodies to the Protein G- and GAM-Modified Particles

A suspension containing 0.035% protein G or GAM-modified particles was prepared in 300 μL of PBS, and then 20 μL of solutions containing the mAbs at 1 mg mL^–1^ in PBS was added. The mixture was incubated on a rotator plate for 1 h at room temperature. Subsequently, the particles were washed once with PBS and the volume was restored to the original 200 μL, yielding a final particle concentration of 0.05% (w/v).

### Inhibition Tests

The inhibition tests were performed in flat-bottomed PS 96-well plates. To do so, 10 μL (0.05%) of functionalized particles was placed into individual wells containing 100 μL of PBS. Next, 5 μL of OTA-F at different concentrations (0.1, 0.01, 0.001, and 0.0001 μM plus a blank control) was added. The same procedure was repeated for a second set of wells, but with the addition of 5 μL of a 1 μM OTA solution. The plate was incubated under gentle shaking for 15 min. The inhibition rate (in percent) was calculated as the quotient of the signals (mean counts in FL1) measured for the wells containing OTA (subscript *x*OTA) and the wells without OTA (subscript *no*OTA), representing the maximum signal, using the same OTA-F concentration in all wells:
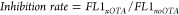


### Competitive Assays

In the competitive assay, 10 μL (0.05%) of particles was dispensed into individual wells of a well plate along with 100 μL of PBS buffer. To initiate the assay, 5 μL of OTA (at different concentrations, including a blank) was added to the wells. Subsequently, 5 μL of OTA-F was introduced into each well, and the assay was gently shaken for 10 min in the dark. Finally, the well plate was subjected to measurement using a flow cytometer with a measurement time of 1 min for a single well.

### Sample Extraction

2 g of sample was placed in a 15 mL tube along with 8 mL of CH_2_Cl_2_ and 200 μL of H_3_PO_4_ 6M. The tube was then placed on a rotator plate for 15 min at 40 rpm and then centrifuged at 8000 rpm for 10 min. From the resulting mixture, 1 mL of the CH_2_Cl_2_ phase was transferred to a 2 mL Eppendorf tube and 500 μL of bicarbonate buffer was added. The Eppendorf tube was mixed on a rotator plate for 10 min, later centrifuged at 6000 rpm for 3 min, and the aqueous phase was collected. Finally, the extract was diluted in assay buffer (PNa75, phosphate buffer pH 7.4, 75 mM) using a dilution factor of 1/25.

### Uncertainty Budget Calculations

The batch-to-batch reproducibility of the synthesized particles is given by the coefficient of variation *C*_V_, as determined via the forward scattering parameter measured with the flow cytometer, resulting in *C*_V_^fsc^= 5% for the PS core, *C*_V_^fsc^= 7% for the particles after the iron oxide coating step and *C*_V_^fsc^= 13% after silica coating.

The coupling of antibodies to SiO_2_@SPION@PS accounts for an additional combined uncertainty of *u*_rel_^*T*1^*=* 3%, including

a) Weighing of 5 mg of the corresponding amino-functionalized particles (balance Mettler Toledo lab 205 ± 0.01 mg) *u*_rel_^a^= 0.2%
b) Dispersing the particles in 500 μL of EtOH after reaction with succinic anhydride (Eppendorf reference pipet 500 μL ± 3 μL) *u*_rel_^b^= 0.6%
c) Diluting 10 μL of the suspension in 100 μL of MES buffer (Eppendorf reference pipettes ±0.1 μL for 10 μL and ±0.8 μL for 100 μL pipet) *u*_rel_^c^= 1.8%
d) Adding 80 μL of a solution of EDC (1%) and 160 μL of a solution of NHS (1%) *u*_rel_^d^=1.3%
e) Adding 50 μL of either GAM or protein G to the solution; *u*_rel_^e^ = 1%
f) Centrifuging and washing (2×) and resuspending in 200 μL PBS; *u*_rel_^f^ = 0.6%
g) Adding 20 μL of the different mAbs *u*_rel_^f^ = 1.5%

The uncertainties of the preparation and execution of the assay include dilutions of the stock solutions of the analyte, i.e., diluting 5 μL of stock solution in 100 μL of buffer (*u*_rel_^V^= 2%), further diluting stock solutions of OTA in water for obtaining standard OTA or OTA-F, in PBS successive dilution of the mother solution: *n* × *u*_rel_^V^, *n*_max_ = 9, mixing of 10 μL of particle suspension (0.05%) and 100 μL of buffer + 5 μL of OTA and 5 μL of OTA-F (*u*_rel_^a^ = 4.8%) and the contributions from the cytometer measurements:

a) Relative uncertainty of counts of particles registered: *u*_rel_^cyt^ ≤ 1.5%
b) Experimental standard deviation for replicate measurements: *u*_rel_^r^ ≤ 2% amounting to a total relative uncertainty of *u*_rel_^T^= 5.8% for the assay.

## Results and Discussion

Our study addresses the application of a next generation of magnetic hybrid microparticles for the determination of small-molecule analytes in rapid cytometry assays using monoclonal antibodies as the detection entity. While the present work demonstrates the performance of the approach with the use case of detecting OTA in wheat flour extracts, the particle architecture already includes a future use of the beads in multiplexed automatized assays. In addition, the aim was to further exploit a straightforward mix-and-read approach as our beads are ideally suited for use with many different antibodies without the need to change the protocol or the beads. Existing magnetic particles face challenges related to stability, functionalization, and weight (sedimentation). To overcome these limitations, we recently improved our promising hybrid particle platform, being advantageous in terms of stability, practical weight, facile functionalization, and customizable surface area,^30,31,35^ with another feature, a magnetic functionality.^34^ The present work reports on the functionalization, deployment, and performance of these beads in analytical assays, offering advantages in terms of efficiency and reliability compared to previous approaches.

**Figure 1:**
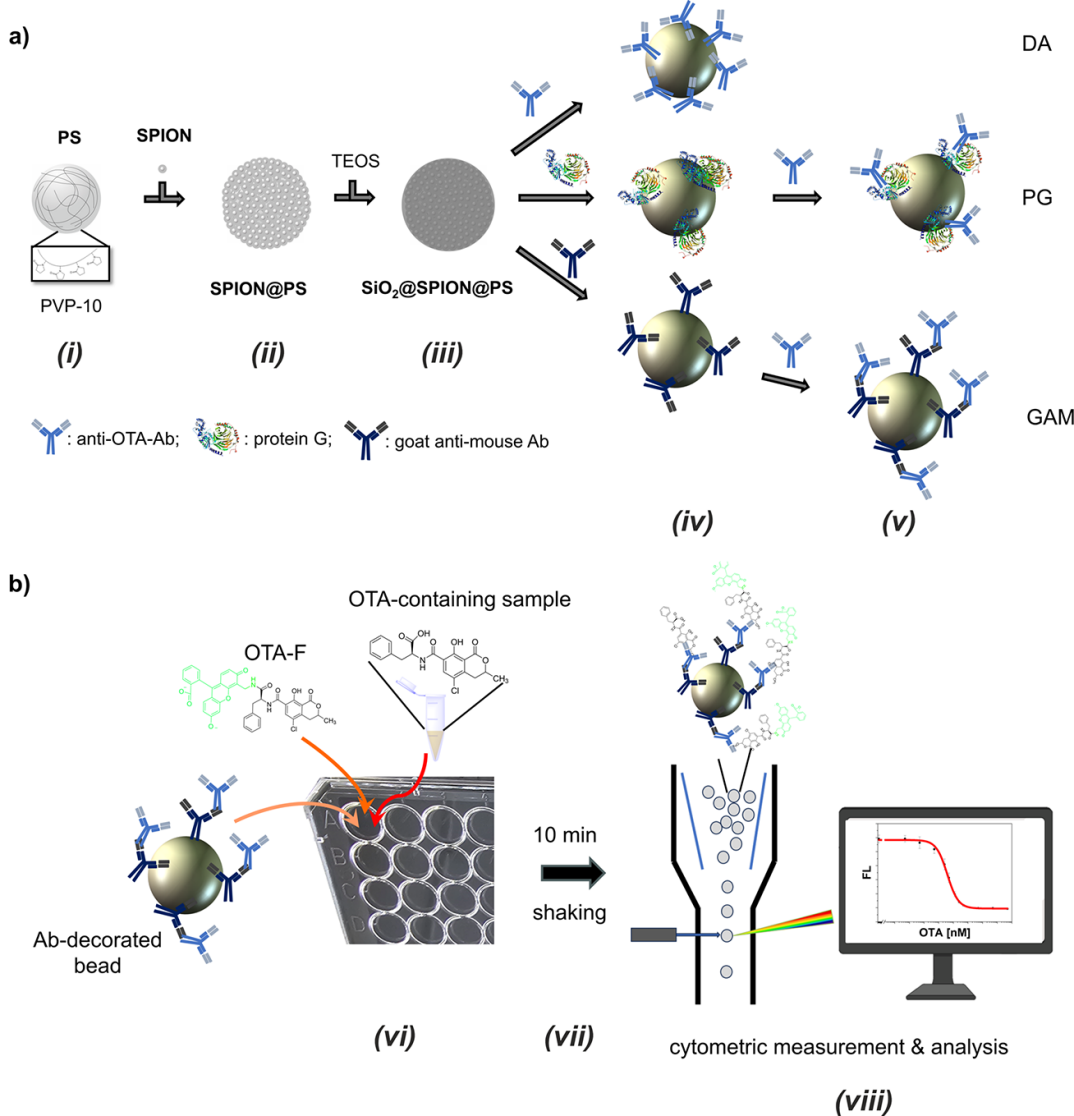
(a) Workflow for bead preparation: coating of PS beads (i) with SPIONs (ii) and an insulating SiO_2_ shell (iii); the amino functionalization and carboxy refunctionalization steps are omitted for simplicity. Binding of antibodies to beads through one-step direct attachment (iv, route DA) or in two steps via protein G (v, route PG) or goat antimouse Ab (v, route GAM). (b) Workflow of mix-and-read assay: pipetting of three solutions into wells (vi), shaking (vii), and cytometric measurement and analysis (viii).

The design rationale of the superparamagnetic core–shell microparticles and their stepwise synthesis is as follows. Poly(vinylpyrrolidone)-stabilized (PVP10, average molecular weight 10 kDa) PS microparticles constitute the core (Figure 1a, (i)), because they can be facilely prepared in sizes well suitable for single-particle assays (1–3 μm) with high monodispersity and the possibility to dope them subsequently with organic dyes via a simple swelling procedure.^31^ A thin layer of SPIONs that does not cover the surface completely is then coated onto the PS cores to endow magnetic properties while avoiding sedimentation issues and leaving space for any optical moieties doped into the core to be excited in an application (Figure 1a, (ii)). Finally, a closed secondary silica shell is coated onto the first shell for protection and further facile functionalization via silane chemistry (Figure 1a, (iii)).

**Figure 2:**
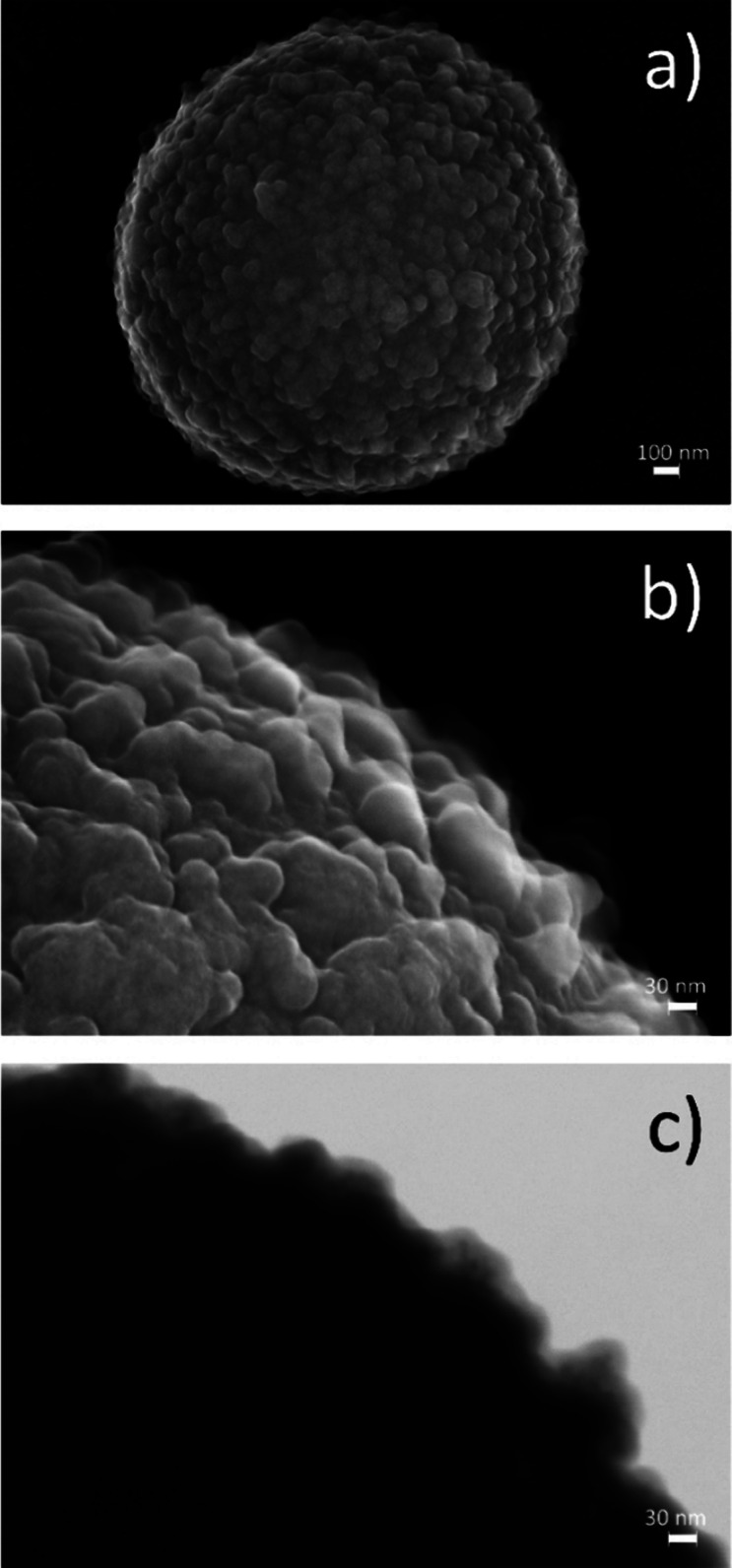
SEM images ((a) full view and (b) close-up of surface) and TSEM images ((c) close-up of surface) of the polystyrene-core magnetic silica-shell beads.

The resulting particles as observed in a scanning electron microscope (SEM) are shown in Figure 2. They have an overall diameter of 1.8 ± 0.1 μm. The silica shell, measuring ca. 30 nm in thickness, incorporates 5 nm SPIONs. The image in Figure 2a shows the whole particle, while the image in Figure 2b highlights the surface area and its roughness. To visualize the SPION layer, TSEM images were taken as seen in the image in Figure 2c. First, these particles have a similar surface area to commercial microparticles for analytical applications (e.g., Dynabeads, Thermo Fisher) while presenting notable advantages: Polyvinylpyrrolidone (PVP) serves as a stabilizer in the particles, not only offering a long-term stability for the SPIONs by themselves^41^ but also facilitating a straightforward decoration with SPIONs before overgrowth of an insulating silica coating. They are lighter in weight than conventional silica or PS particles containing the magnetic iron oxide nanoparticles in the microbead’s core,^10,12^ making them more suitable for longer use in various applications. The particles’ distinctively textured, high-surface area structure can be attributed to the presence of SPIONs partially decorating the pure PS core. This layer, combined with a dense silica coating, results in a rougher surface, as previously described.^34^

An important new aspect of these particles is this type of layer formed by the SPIONs. Although they are not magnetic outside a magnetic field due to the size of the nanoparticles, the entire core–shell particle can easily be moved by a magnet. The presence of a rather loose layer instead of a closed shell of nanoparticles also enables optical encoding of the cores, since a dense shell of nanoparticles would result in optically opaque core particles.^35^

Lastly, the silica coating not only provides multiple avenues for functionalization but also acts as a protective barrier, enabling the particles to be used in acidic environments where iron would typically oxidize and lose its magnetic properties. In our case, this silica outer shell was employed to include amino groups through classical silane chemistry using APTES. These amino groups were later converted into carboxylic acid groups, via succinic anhydride, for the further immobilization of the proteins.

**Figure 3:**
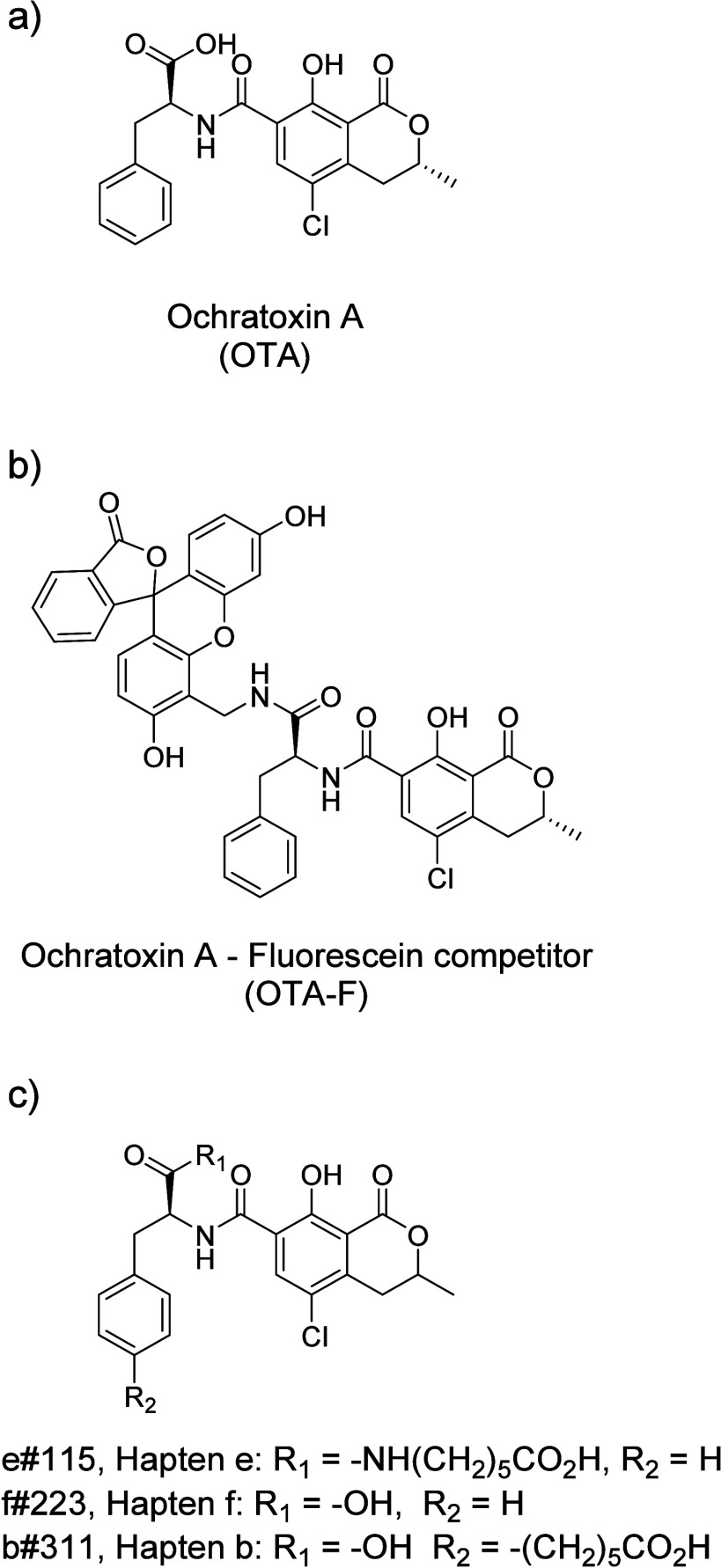
Structures of (a) OTA, (b) fluorescent competitor OTA-F, and (c) haptens used for antibody generation.

To develop an easy-to-handle assay protocol using the superparamagnetic hybrid PS-core magnetic silica-shell beads, several tests were conducted. These tests included the binding of different proteins, such as primary monoclonal antibody, secondary polyclonal antibody, or protein G to the particle surface (Figure 1a, (iv) and/or (v)), as well as the investigation of antibody interactions with the selected fluorescent competitor and the target analyte, OTA. Considering our previous experience working with this mycotoxin, three different monoclonal anti-OTA antibodies were selected for evaluation (e#115, f#223, and b#311; labeling follows the labeling in ref (40)). These three antibodies were selected because they were able to interact with a competitor for OTA prepared through the same position as the fluorescent fluorescein-containing competitor (OTA-F) selected for this study. Figure 3 shows the structure of OTA, OTA-F, and the haptens that were previously used for antibody generation. Each of the haptens was used for the generation of one of the antibodies tested here, as indicated by the haptens and the letter in the antibody code.

To bind the different proteins to the particle surface, we used EDC/sNHS chemistry to form covalent amide bonds between the amino groups of the lysine residues of the proteins with the carboxylic acid groups on the surface of the particles. Additionally, the purpose of using protein G (Figure 1a, route PG) or GAM (Figure 1a, route GAM) as assisting capture proteins was to evaluate the importance of the orientation of the primary anti-OTA antibody. This was done with the aim to minimize statistical errors, ensuring that the primary antibody would consistently have the correct orientation (paratope facing the analyte) on the particles. As a control, the primary anti-OTA antibodies were also directly attached (Figure 1a, route DA) to the surface using the same EDC/sNHS approach. Considering the final particle assay application, flow cytometry was chosen as the preferred method for all testing due to its user-friendly features, including the availability of an autosampler and rapid assay readout (Figure 1b).

The fluorescence emitted by the OTA-F competitor on the surface of the particle is centered at 518 nm and was detected through a 533/30.H bandpass filter before correlation with the FSC signal to distinguish it from the excess of competitor still in solution.

In a preliminary study, to test the capability of these antibodies to recognize the OTA-F competitor, three immobilization approaches via routes DA, PG, and GAM were evaluated for the three anti-OTA antibodies. The maximum signal from the surface of a single particle was measured in the absence of OTA (most OTA-F bound), while also a pronounced inhibition rate was assessed for a relatively high concentration of OTA (40 nM), carrying out these experiments for all combinations. The inhibition rate is defined here as the quotient of the signals in the wells with OTA and the signals in the wells without OTA, with all wells having the same OTA-F concentration; see Materials and Methods for details.

**Figure 4:**
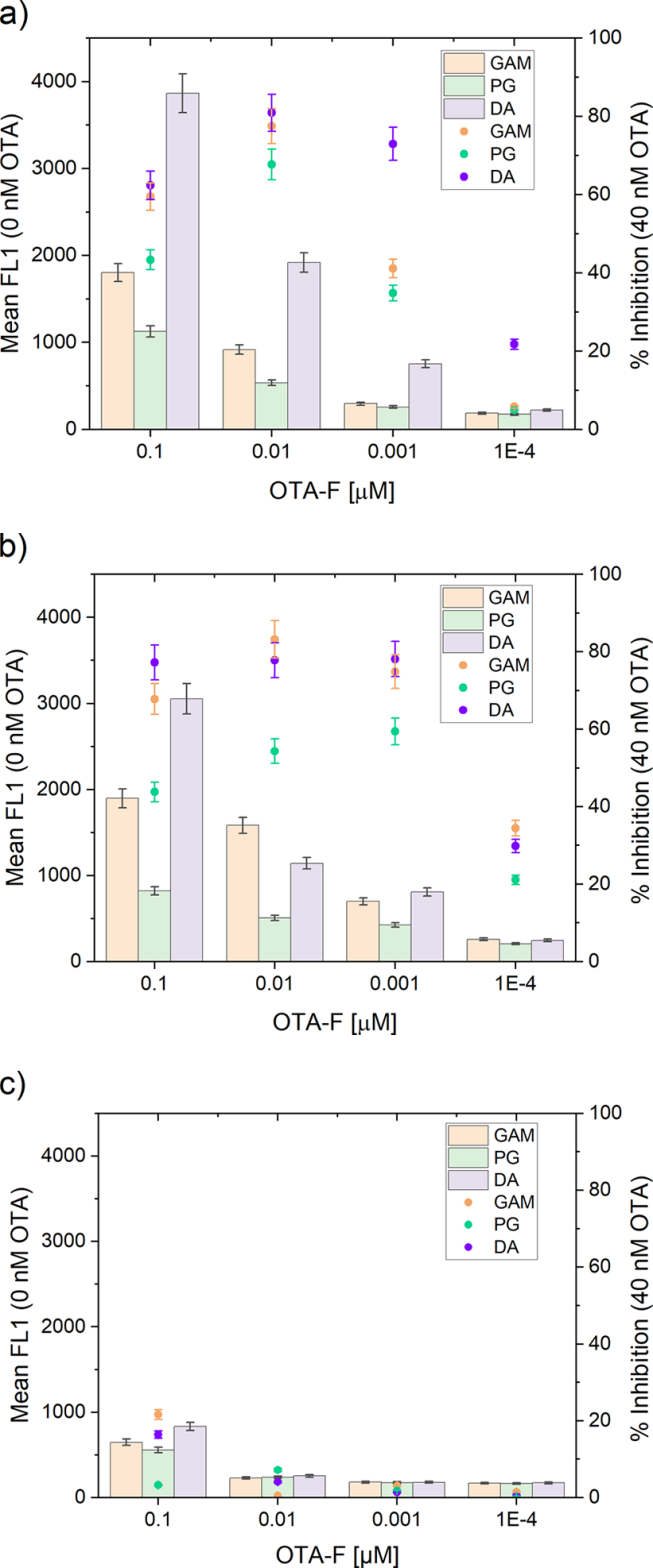
Maximum signal of the bound competitor in the absence of analyte in the assay (left *y*-axis) and inhibition rate for a concentration of 40 nM of OTA (right *y*-axis) for (a) antibody e#115, (b) antibody f#233, and (c) antibody b#311 attached via routes DA, PG, and GAM to the title beads.

At this point, the concentration of antibody was kept constant at 300 ng of antibody per μg of particle. Figure 4 shows the graphs representing the inhibition rates as dotted lines and the maximum signals as bars for the nine types of particles used in the study. The left *y*-axis denotes the maximum signal achievable with the competitor for each type of binding, while the right *y*-axis represents the inhibition of the competitor in the presence of 40 nM OTA. To ensure the lowest rate of false-negative results, it was crucial to employ the lowest possible concentration of competitor. Excess fluorescence from the leftover competitor could potentially lead to inaccurate outcomes via unspecific binding. The highest inhibitions were observed when antibody e#115 was directly bound to the particles (route DA, 80%) or when antibody f#223 was used in conjunction with GAM (route GAM, 85%). Conversely, these testing steps revealed that antibody b#311 showed no significant interaction with the competitor, probably due to the lack of a spatial linker between the OTA and fluorescein subunits, as reporters with longer linkers were able to interact with this antibody in ELISA.^21^ Therefore, antibody b#311 was excluded from further investigations here.

To determine the optimal signal-to-noise ratio, we evaluated different concentrations of competitor with the other two antibodies. The best ratio was achieved with a concentration of 0.01 μM of OTA-F. While antibody e#115 displayed the highest overall signal, its inhibition rate reached 80% in the case of antibody binding along route DA. On the other hand, antibody f#223 exhibited successful performance with the secondary antibody attachment via route GAM and an improved inhibition rate of 84%, aligning with the objective of this project. Consequently, further testing involved a comparison of e#115/DA and f#223/GAM.

To gain more insight into the antibody immobilization step, we decided to determine the required amount of antibody for maximum coverage of the particles. In that regard, the assay with antibody e#115 was chosen for this evaluation since it was performing better in the DA approach.

To account for any uncertainties regarding the behavior of the antibody on the surface, ideally full functionalization of the surface is desirable. Because of that, we conducted further tests using two different concentrations of this antibody: 300 ng of antibody μg^–1^ of particle, a similar concentration to the one employed in the preliminary tests, and 450 ng of antibody μg^–1^ of particle.

**Figure 5:**
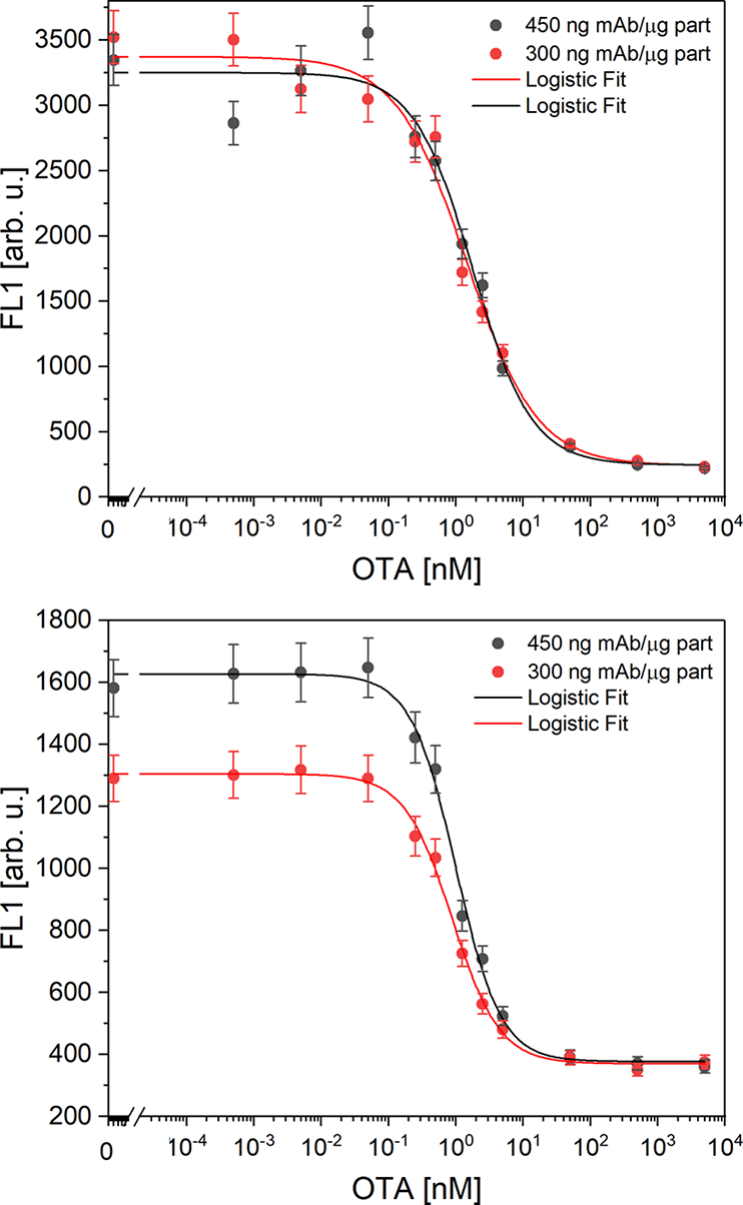
Calibration curves using e#115/DA (top) and f#223/GAM (bottom) in different amounts to determine the maximum coverage of the particles.

The calibration curves were generated by testing different concentrations of OTA in the presence of a 0.01 μM competitor concentration. Figure 5 (bottom) shows that both concentrations ultimately yielded similar signal intensities, indicating that the maximum binding capacity of the particles’ surface had been reached with the lower concentration tested. The half inhibitory concentration (IC_50_) commonly identified as the concentrations at the inflection point when the minimal asymptote tends to zero for these two assays were determined to be 1.4 and 1.7 nM for the concentrations of 300 and 450 ng of antibody μg^–1^ of particle, respectively.

Considering the similar behavior of both concentrations shown, the lowest one was selected for further optimization. Since antibody f#223 was showing a better inhibition in the preliminary test when immobilized via route GAM, this assay was chosen for further development. In the same way as before, two different antibody concentrations were tested, 300 and 450 ng of antibody μg^–1^ of particle.

At this point, it must be noted that the particles were previously functionalized with a large excess of capture antibody GAM (960 ng of secondary antibody μg^–1^ of particle) to saturate the particle surface and eliminate the availability of active places as a limiting factor. Figure 5 (top) presents a signal increase with the amount of primary antibody f#223 used, suggesting that the amount of capture antibody is adequate for the tested concentrations of monoclonal antibody.

Therefore, the decisive factor in selecting the optimal assay was a compromise between an adequate maximal signal and minimal IC_50_. The IC_50_ values for the two antibody concentrations tested, 300 and 450 ng of antibody μg^–1^ of particle, were 0.7 and 1.0 nM, respectively. The fact that the maximum specific binding (*B*_max_) and IC_50_ are differently affected by a change in antibody concentration for route GAM compared to route DA, i.e., a reduction in *B*_max_ with similar IC_50_ for route GAM compared to similar *B*_max_ and IC_50_ for route DA, is presumably due to the different ways of antibody attachment. As mentioned above, antibodies e#115 are covalently anchored to the surface of the beads in route DA and both concentrations resulted in a virtually identical functionalization density, leading to average FL1 signals of 3310 ± 60 counts and a 20% higher IC_50_ for the higher antibody concentration. However, with route GAM, two biomacromolecules are sequentially bound to the surface of the beads, so that not only the covalent functionalization density with GAM is decisive, but also the noncovalent binding of f#223 to GAM plays a role. Obviously, the occupation of the GAM units by f#223 is not yet quantitative at the lower concentration, which leads to a 25% higher *B*_max_ at the 450 ng of antibody μg^–1^ of particle. In addition, IC_50_ is also 45% higher for the higher antibody concentration.

As lower IC_50_ values correspond to improved detection limits and the absolute signal intensities measured are sufficient for all assay combinations, the optimized assay utilized the lower antibody amount of 300 ng of antibody μg^–1^ of particle.

We made additional efforts to enhance the assays by experimenting with postantibody attachment washing steps and modifying the buffer composition. However, these attempts yielded no discernible improvement.

The introduction of an additional washing step with buffer, in addition to centrifugation and solvent exchange, did not lead to any noticeable changes in assay signals. Similarly, transitioning from a PBS to a TRIS-based buffer did not result in an increase or decrease in signal intensity beyond measurement uncertainty.

Thus, advantageously, the mix-and-read approach as shown in Figure 1b proved to yield optimum performance while being simple and fast.

**Figure 6:**
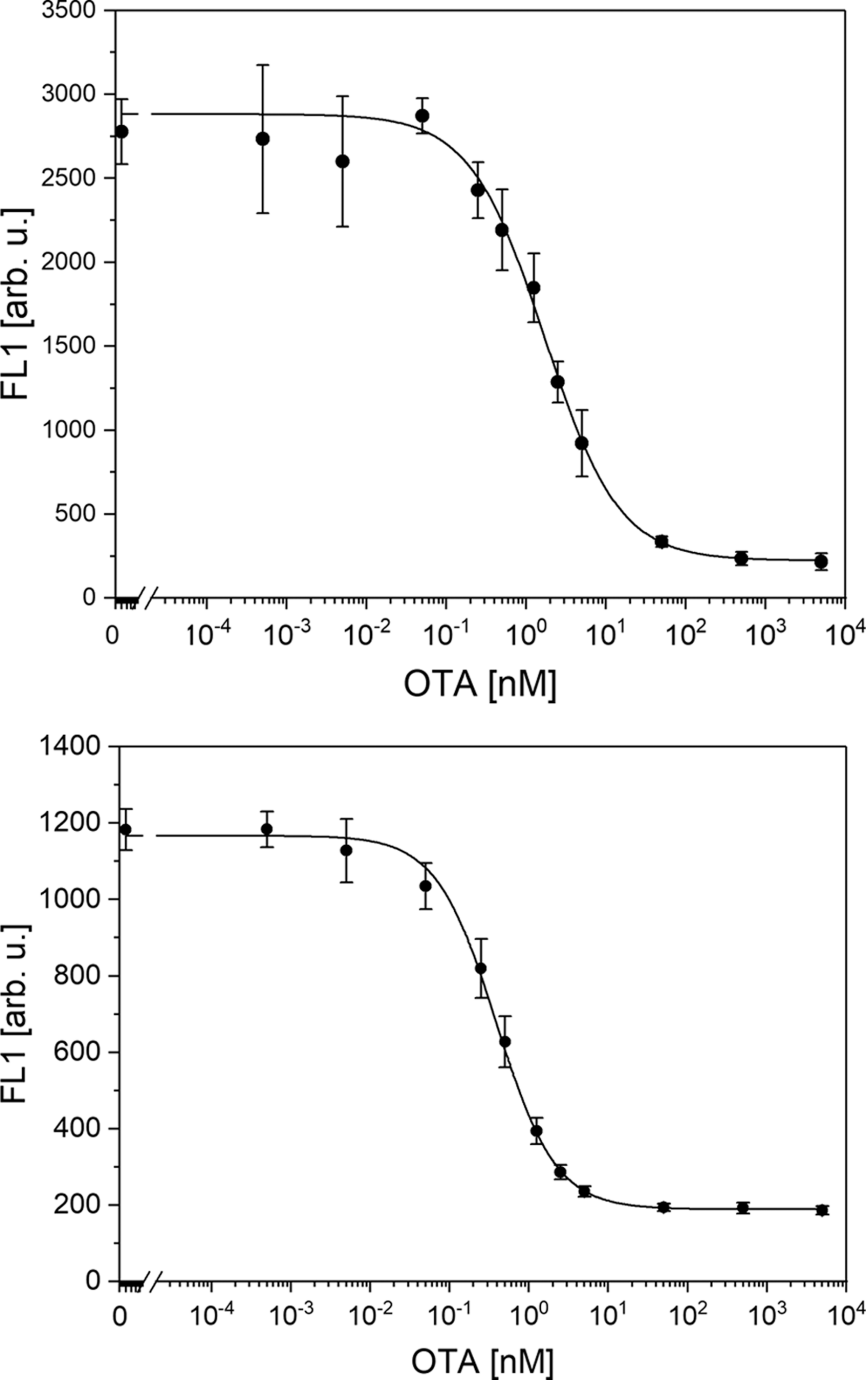
Calibration curves of the final optimized assays; assay based on combination antibody e#115/DA (top) and assay based on combination antibody f#223/GAM (bottom).

To assess the reproducibility of the developed assay, its day-to-day performance was studied. The assay was performed on four separate days, with four different batches of beads, with three replicates each, yielding the calibration curves presented in Figure 6.

Figure 6 provides a direct comparison of the two particle-based immunoassays, i.e., in the top panel, OTA was again detected with antibody e#115 attached to the surface of the particles along route DA, while in the bottom panel, the combination f#223/GAM was used. The figure shows that the detection limits exhibited minimal variability throughout the 4-day testing period, and with an IC_50_ of 0.1 nM, the assay involving the use of GAM and monoclonal antibody f#223 appears to be the most effective.

The larger error observed in particles using antibody e#115 is likely due to the direct binding method used in their preparation. Without control over the orientation of the paratope region, the uncertainty in measuring lower concentrations of OTA is mainly influenced by random errors due to the lack in directionality rather than the preparation of the carrier particles or the assay protocol.

The limit of detection (LoD, according to DIN 32645:2008-11) achieved by our assay using the secondary antibody is 0.025 nM, which is in the lower range of cytometry assays for OTA reported in the literature.^26−29^ An essential improvement in comparison to commercial assays is the overall assay time. The entire process takes less than 15 min, 10 min for incubation plus transfer time to the flow cytometer, and ca. 1 min measurement time for a cytometry run, in contrast to 1 h or more for most reported examples,^4−7,10,12^ the main improvement being the simplicity and greatly reduced incubation time.

The ability to detect low concentrations of the target analyte further demonstrates the robustness and reliability of our developed assay. Notably, the achieved detection limit is comparable to that of many immunochemical methods, such as ELISA, and even outperforms certain techniques like electrochemical analysis.^21,42,43^

The relatively low and consistent error observed in our measurements ensures compliance with the mycotoxin testing limits set by the European Union (EU).^20^

The versatility of the analytical method described in this study allows for its applicability in various particle-based assays, only limited by the availability of a competitor and its performance with the available antibodies.

For further proof, we conducted a real-life example by testing wheat flour obtained from a local mill. The flour sample was obtained directly from the mill without any further processing, reflecting the typical handling at the facility. To ensure reliable measurements, a portion of the sample was spiked with OTA. The initial concentration of OTA was known through a regular analysis by Eurofins, commissioned by the mill.

We spiked the samples with an OTA standard, resulting in a concentration of 6.1 μg kg^–1^, while the pure flour sample contained 0.2 μg kg^–1^, a value below the maximum limit established by the EU.^20^ Following an extraction method previously described based on the acidic characteristics of OTA,^21^ we performed the assay and obtained values of 0.4 ± 0.1 μg kg^–1^ for the blank flour sample and 6.4 ± 0.1 μg kg^–1^ for the spiked sample. This resulted in a recovery rate of 104%, which falls within the commonly accepted range of 90–110%.

We attempted alternative extraction methods, including the use of ethanol as the matrix, but unfortunately, these efforts did not yield any meaningful results. Using ethanol only in the extraction step, no signal was detected, and when measurements were conducted in ethanol, a matrix effect was observed, ultimately resulting in inconclusive outcomes.

The successful recovery demonstrates the accuracy and efficiency of this assay and further highlights its suitability for practical applications. Additionally, the speed of the assay shows by requiring only a 10 min incubation followed by measurements completed in less than 30 s.

The assay presented in our study demonstrates a competitive performance for OTA detection compared to existing assays, in terms of both detection limit and analysis time. The total time required for our assay is less than 11 min per sample, which is on par with the performance of LFIAs.^21,22,44,45^ Unlike LFIAs, which typically cannot perform parallel analyses, our assay utilizes a well plate autosampler with a cytometer, allowing for rapid sequential analysis. This setup provides a significant advantage in terms of throughput and speed and has the advantage of having much better multiplexing potential than LFIAs due to the PS core, which can be easily encoded with dyes.

While ELISAs generally offer the capability to process multiple samples simultaneously, making them suitable for high-throughput scenarios, they are commonly characterized by significantly longer analysis times while showing comparable detection limits.^21,46,47^ Also, the use of other binders such as nanobodies^48,49^ or aptamers^50^ or the employment of novel amplification strategies^51−53^ has not changed the situation with respect to assay time and has also not led to dramatic improvements in detection limits. Compared with ELISAs, our assay offers a much faster alternative without sacrificing sensitivity. The assay developed here is especially advantageous in situations requiring immediate decision-making, like the processing of a shipment in a mill. It would also be beneficial in laboratories where the throughput of LFIA is needed with higher sensitivity and specificity.

With the ease and speed of this assay, further work in our laboratories addresses multiplexed analysis, allowing for the detection of multiple toxins simultaneously.

## Conclusions

Our present work focused on the development and optimization of a particle-based immunoassay for the detection of ochratoxin A. By employing novel particles with improved stability, easy functionalization, and low practical weight, we were able to overcome the limitations of existing magnetic particles and develop a fast mix-and-read immunoassay format with an overall assay time of <15 min.

Flow cytometry proved to be a suitable method for the measurement of the assay, offering user-friendly handling and quick readout. The calibration curves obtained through the assay demonstrated the maximum binding capacity of the particles and the impact of different antibody attachment and concentrations. Day-to-day measurements showed consistent calibration curves with minimal variation especially for the route employing a secondary goat-antimouse antibody for the decoration of the particles with the primary antibody.

Notably, the limit of detection for the final optimized assay, using the secondary antibody, was determined to be 0.025 nM, surpassing the detection limits of many existing methods, including electrochemical ones.

Furthermore, the developed assay demonstrated its practicality and applicability in a real-life scenario by successfully analyzing a flour sample obtained from a mill and detecting OTA with an excellent recovery rate. This underscores the ease and efficiency of the proposed assay, making it a valuable tool for rapid mycotoxin testing in various food industries.

With its versatility, potential for multiplexing, and suitability for simplified fluidic systems due to the magnetic properties of the particles employed, this assay holds promise for the detection of other toxins or low molecular weight pollutants as well. The results obtained in this study contribute to the advancement of mycotoxin detection methods and offer practical solutions for food safety analysis.
